# What families really think about the quality of early intervention centers: a perspective from mixed methods

**DOI:** 10.7717/peerj.10193

**Published:** 2020-10-20

**Authors:** Rita Pilar Romero-Galisteo, Pablo Gálvez Ruiz, Angel Blanco Villaseñor, Maria Rodríguez-Bailón, Manuel González-Sánchez

**Affiliations:** 1 Department of Physiotherapy, University of Málaga, Málaga, Spain; 2 Valencian International University, Valencia, Spain; 3 Department of Social Psychology and Quantitative Psychology, University of Barcelona, Barcelona, Spain

**Keywords:** Early intervention, Family, Health care, Quality assessment, Mixed methods

## Abstract

**Background:**

Families are a fundamental aspect in the current perspective of Early Intervention, and knowing their opinion with quantitative and qualitative research is necessary for its improvement. The objective of this research was to evaluate the quality of the service perceived in Early Intervention Centers and its relationship with satisfaction and future intention, as well as to identify factors that are associated with the perception of users.

**Methods:**

A measurement model of 50 items and an open question to gather qualitative information was used in a sample of 233 participants. A confirmatory factor analysis and a regression analysis were conducted. Regarding the qualitative data, the information was subjected to a thematic content analysis in order to delve into the perception of the participants.

**Results:**

The model showed a satisfactory fit and the regression analysis indicated that treatment rooms (*β* =  − 0.28) and adaptation of activities (*β* = 0.27) have greater weight with respect to satisfaction, whereas for future intention, the factors of greater weight were adaptation of activities (*β* = 0.23) and location (*β* = 0.20). The qualitative analysis showed three themes: facilitators, barriers and suggestions for improvement. Within facilitators, the participants were satisfied with the Early Intervention professionals, and they made improvement suggestions for the detected barriers to improve the facilities and the follow-up of the child.

**Conclusions:**

The study offers a wide perspective of the perception of the service with an active participation of families in the treatment within the Early Intervention service. This will allow professionals in Early Intervention, service providers and researchers to consider the families as intervention agents capable of providing their opinion and making decisions, and not only as passive elements.

## Introduction

Early Intervention Service (EIS) is provided to children from birth to 6 years of age who suffer from any type of disorder or are at risk of developing one (GAT, 2005), a definition that is in line with the one established by Guralnick (2015), with a child-centered approach and aimed at the rehabilitation of the child and the family where professionals adopt most of the decisions on all aspects of the EIS process (Serrano et al., 2017). Early childhood intervention locally, nationally and internationally has been found to be effective in improving child, parent and family outcomes (Guralnick, 1997) and generating benefits to society far beyond program or intervention costs, which may include a decrease in welfare spending, as well as a reduction in the costs of education and health services programs (Sukkar, Dunst & Kirkby, 2017). In this sense, a well-designed and properly implemented EIS cannot be considered as a mere consumer of resources. A correct intervention can prevent future problems for the child and his/her family, and save resources for society (Cayo, 2005). In fact, as stated in the Report on Early Childhood in Spain (González-Bueno & Bello, 2014), intervening in early childhood is fair and cost-effective, it benefits everyone and it is a fundamental element in complying with the rights of children and transforming societies.

Nowadays, society has shifted from a model focused on the child and his/her rehabilitation, to a model in which interaction with the family is considered fundamental (Andrés & López, 2012; Bricker, Xie & Bohjanen, 2018), including the latter in a more or less systematic manner in the work methodology of EIS. This model requires fluid and positive communication between families and professionals from the beginning of the interaction in order to evaluate important matters in EIS. Analysing such information will allow professionals to develop an intervention that grants families some degree of responsibility with the service they are receiving. Therefore, an active demand of the families to improve the quality of the provided EIS is essential and, indirectly, improves its efficacy (Verdugo, 2005). Therefore, further studies should combine the strengths of both quantitative and qualitative analyses, which would allow exploring this field with the aim of obtaining a more complete view of this phenomenon (Suri, 2013).

### Literature review

Service quality is understood as the mutual relationship of satisfaction and expectations between a consumer and the organization that attends to his/her needs (Duggirala, Rajendran & Anantharaman, 2008). In the case of EIS, the European Foundation for Quality Model is taken as a reference, although different authors have stated that it is a model that does not consider important aspects related to the characteristics of EIS (Shonkoff & Levitt, 2010; Bruder & Dunst, 2015) and its quantification is still a difficult task in the present (Jemes et al., 2019). In fact, and despite the importance of evaluating the quality of EIS for administrators, professionals and users, there is no consensus about the dimensions of its composition (Polyakova & Mirza, 2016).

Research on customer behavior considers the quality of services and satisfaction the previous step to fidelity or loyalty (Bakker & Crompton, 2000). Satisfaction has been widely studied in the service sector and its interest is increasing in the scientific literature, as it is considered a precursor of loyalty (Oliver, 1999; Caruana, 2002; Olsen, 2002). If a consumer is satisfied, the services or products provided are more likely to be used or consumed again (Bernhardt, Donthu & Kennett, 2000), and the consumers are more likely to recommend these to other people (Zairi, 2000). Therefore, considering the above mentioned, a satisfied consumer is more likely to have a positive perception of the organization and also to be more loyal to it (García-Fernández et al., 2018a), thus creating a strong relationship between these variables. In the scope of EIS, few studies have specifically analysed the satisfaction of families (García et al., 2008). However, the studies conducted in Spain with EIS-user families show a positive valuation of these services and great satisfaction (Giné, 2002), which is consistent with the results obtained from a sample of people with intellectual disabilities who are users of organizations linked to the *Plena Inclusión* (full inclusion) *Confederation* (Gracia, Vidal-Sellés & Martínez-Tur, 2017).

However, future intention is a construct that has not been used in studies focused on EIS, despite the fact that the strong and positive relationship between this variable and satisfaction has been widely studied in other disciplines (Anderson & Fornell, 2000; Carlson & O’Cass, 2010; García-Fernández et al., 2018b). In this type of service (EIS), the families can always request to voluntarily withdraw from the center or be transferred to a different one, and the corresponding administration must allow these situations when there are justified reasons (e.g., change of family address).

Therefore, with the aim of evaluating the perception toward the service offered to families attended to in EI centers, we adopted an approach combining quantitative and qualitative analyses to obtain detailed information about the perception of experiences from different perspectives. We used a measuring model that includes the dimensions of service quality, satisfaction and future intention. In addition, this model gathers and analyses information through an open question with the aim of identifying information that could not be captured in the items of the questionnaire about the daily situations related to the service received in EIS. Mixed methods are used with increasing frequency in Health Sciences (Plano-Clark, 2010) to obtain a more complete understanding of problems and to know the opinions of the participants (Guetterman, Fetters & Creswell, 2015). Thus, it is a research methodology that compiles and analyzes both quantitative and qualitative data, making use of the advantages of both approaches (Creswell, 2015). In this sense, quantitative data provide a general view of the research problem, whereas qualitative data allow explaining the quantitative results more accurately, as they make it possible to explore the different opinions expressed by the participants.

## Materials & Methods

### Participants and procedures

A mixed-methods design was employed; quantitative and qualitative data were collected to measure participants’ service perceived in Early Intervention Centers. We used a sequential explanatory method in which qualitative data supported quantitative data and enabled better understanding of the results. The convenience sample of the study consisted of a total of 233 parents of children who receive treatment sessions in two Early Intervention (EI) centers located in Málaga (Andalusia, south of Spain). The sample size with usable data needed to estimate percentages so that their 95% confidence intervals were no wider than ±5% while allowing for the total finite population size of the two EI centers (*N* = 301) would be 170, and, after allowing for approximately one-quarter of questionnaires not being completed, this *n* = 233 would be sufficient. Only one questionnaire was given per family, and the participants answered the different questions in the waiting room of the EIS center in the presence of a researcher to resolve any uncertainties for the participants but without influencing the answers, respecting at all times the voluntary nature of participation. None of the families refused to participate and all of them met the inclusion and exclusion criteria established. Regarding inclusion, the recruitment of participants was focused on (1) families with children aged between 1 and 6 years, and (2) families with children attended to in EI centers for a minimum time of 6 months. The study excluded those who had been receiving treatment in the EI center for less than 6 months, those who were not the usual relative accompanying the child to the EIS center, and those who could not provide a proper open answer in the questionnaire due to problems derived from poor comprehension or deficient written expression. The questionnaires took about 10–12 min to complete.

This study was approved by the Ethics Committee of the University of Malaga (code: 32-2017-H). The authors informed the participants about the objectives, the use of the information that would be generated, the voluntary nature of participating in the research, and the confidentiality of the answers. All participants signed an informed consent before the collection of data, in compliance with the Declaration of Helsinki. The data were gathered between May and June of 2017 directly in the EI centers and no incentives were offered for participating in the study.

### Measures

#### Quantitative data collection

Data were gathered about the following specific individual characteristics: age, gender, academic studies, family relationship, and time since the beginning of the EIS treatment received.

The participants completed in writing and face to face the Inventory of Quality in Early Intervention Centers (IQEIC) (Romero-Galisteo, Morales-Sánchez & Hernández-Mendo, 2015). This questionnaire consists of 48 items, and following the original scale, the response option of each item was based on a 5-point range (1: totally disagree with the content of the item –5: totally agree with the content of the item), and it is composed of six subscales and fourteen factors: facilities of the center (F), treatment rooms and material (TRM), specific sessions (SS), specialized staff (SP), general information, and technical assistance (TA), which showed acceptable values of internal consistency (*α*F = 0.88; *α*TRM = 0.93; *α*SS = 0.77; *α*SP = 0.92; *α*TA = 0.85). For the present study, the general information subscale was excluded, mainly due to the fact that it contained irrelevant and anachronistic information for this research, since the ways to access EI centers have been modified by the public administration and currently this can only be achieved with a prescription from health professionals. Furthermore, it is mandatory for every healthcare service to have mechanisms for the management of possible enquiries, suggestions and complaints. Thus, a final questionnaire (Table 1) of 43 items with a twelve-factor structure was used. A scale with 3 items was used to evaluate the satisfaction (Oliver, 1997) obtaining an internal consistency of *α* = 0.90, and a scale of 4 items was adopted to evaluate future intention (Zeithaml, Berry & Parasuraman, 1996), whose internal consistency was *α* = 0.90.

**Table 1:** Dimensions, factors and number of items of the evaluation instrument used.

Dimensions	Factors	Number of items
Facilities	Location	3
	Environmental conditions	6
	Waiting room	4
Treatment rooms and material	Treatment rooms	4
	Material	5
Specific sessions	Consumer service	3
	Schedule adaptation	2
	Frequency adaptation	2
Specialized staff	Qualification and distance	5
	Personnel coordination	3
Technical assistance	Adaptation of activities	3
	Technical information	3
Satisfaction		3
Future intention		4

#### Qualitative data collection

This study employed a qualitative approach using the descriptive phenomenological method, whose main purpose is to study the daily personal experience of the participants, understood as non-conceptualized or categorized experience (Báez, 2014; Creswell, 2007). Thus, we used a non-experimental parallel design, conducting a single measuring of the recruited sample from the qualitative perspective, adjusted to the objective of the study (Ballester, 2001), since it was the reality described by the participants (Patton, 2015). After the participants answered the questionnaire items, the authors asked an open question to the participants: “How would you value the EI center? Please, include suggestions about each of the following aspects”. To ensure the validity of the content, a board of three experts approved the answers by indicating that these could contain information about each of the subscales of the questionnaire. The board of experts was formed by a psychologist, a physiotherapist and a speech therapist specialized in EIS; in fact, these three disciplines are the ones that must make up the basic multidisciplinary team that the public administration demands to constitute an EI center in Andalusia (Southern region of Spain), in compliance with article 4 of the Law of December 13th of 2016.

### Data analysis

With respect to the quantitative data, a descriptive analysis of the demographic characteristics of the sample was conducted (age, gender, academic studies, family relationship, and time since the beginning of the EIS treatment received). The evaluation instrument composed of the IQEIC (Romero-Galisteo, Morales-Sánchez & Hernández-Mendo, 2015) and measures for satisfaction (Oliver, 1997) and future intention (Zeithaml, Berry & Parasuraman, 1996) was subjected to a confirmatory factor analysis, using the following goodness-of-fit indices and the maximum likelihood method: *χ*^2^/gl (<3) (Kline, 2005), comparative fit index (CFI) and incremental fit index (IFI) (>0.90) (Hair et al., 2009), residual root mean square (RMR) less than 0.05 and root mean square error of approximation (RMSEA) between 0.05 and 0.08 (Marsh, Balla & Hau, 1996), with the respective 90% CI. For the factorial weights of the items, the values above 0.40 were considered adequate (Hair et al., 2006). The multiple regression analysis is one of the methods to describe the relationships between one dependent variable and multiple independent variables, and in this study was conducted to determine the predictive capacity of the model about satisfaction and future intention (dependent variables) for the 12 factors provided in the IQEIC instrument (independent variables). The assumptions of normality and homoscedasticity were verified through the Kolmogorov–Smirnov test (*p* > 0.05) and Levene’s test (*p* > 0.05). For each regression model, the coefficient of determination (R^2^; it represents the proportion of variance of the dependent variable that is explained by the independent variables), and the variance inflation factor (VIF; values above 10 indicate problems of collinearity (Gujarati & Porter, 2009) was calculated. The statistical analyses were conducted using SPSS and AMOS v.21.0. With respect to the qualitative data, the answers to the open question were subjected to thematic content analysis, following Leuven’s Guide for Qualitative Data Analysis (QUAGOL) (Dierckx et al., 2012), which comprises two parts: (1) a thorough preparation of the coding process, and (2) the real coding process using a specific qualitative software (Atlas.ti v.7.5.10). Three researchers went through the transcriptions independently and generated units of analysis using an inductive and deductive approach. The researchers carried out the analysis by comparing the interpretations of the individual analyses, while trying to reach a consensus. The differences between the researchers were resolved through discussion. This software allows organising the comments provided through the identification of different sub-categories to classify and group the perceptions expressed by the participants, facilitating the consequent analysis.

## Results

**Figure 1:**
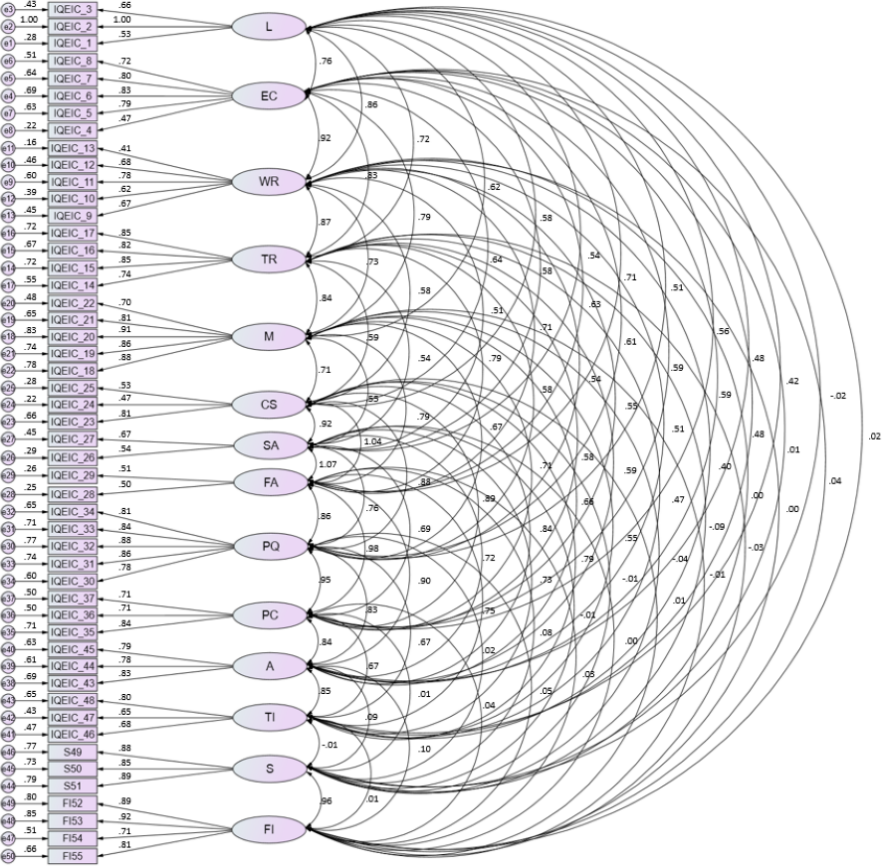
Confirmatory Factor Analysis of IQEIC. IQEIC, Inventory of Quality in Early Intervention Centers; L, Location; EC, Environmental conditions; WR, Waiting room; TR, Treatment rooms; M, Material; CS, Consumer service; SA, Schedule adaptation; FA, Frequency adaptation; PQ, Personnel coordination; A, Adaptation of activities; TI, Technical information; S, Satisfaction; IF, Future intention.

The descriptive statistics of the participants are shown in Table 2, most of whom were women (70.8%). Regarding age, 55.4% of them were between 31 and 40 years old, 19.7% between 21 and 30 years old and 15.9% between 41 and 50 years old, whereas those under 20 years of age and over 51 represented 1.7% each (5.2% missing). With respect to the relationship with the child attended to in the EI center, 66.5% were mothers and 25.3% fathers, and the education level of the participants was mostly primary (33.9%) and secondary (35.6%); 54.9% of the participants have used the service for less than one year. The IQEIC measuring model, satisfaction and future intention revealed an adequate fit: [ *χ*^2^(1080) = 1940.48 (*p* < .001); *χ*^2^/gl = 1.79; CFI = 0.91; IFI = 0.90; RMR = 0.042; RMSEA = 0.059 (90% CI= 0.54, 0.63)]. All factor loadings are over 0.40, ranging from 0.41 to 0.99, indicating that each item is appropriately captured in its respective factor (Fig. 1). For the regression model, satisfaction and future intention were used as dependent variables, whereas the factors of the IQEIC were selected as the independent variables, with the aim of identifying the variable with the highest predictive capacity for each model. Despite rejecting the assumption of normality in the two models (*p* < 0.05), homoscedasticity, collinearity and the absence of extreme values are confirmed. The analyses indicated that the model to explain satisfaction (*R*^2^ = 0.10; VIF = 2.083) has the predictors “adaptation of activities” (*β* = 0.27, *p* = 0.020) and “treatment rooms” (*β* =  − 0.28, *p* = 0.026) as the only factors with significance, whereas in the model to explain future intention (*R*^2^ = 0.17; VIF = 2.000), in addition to “adaptation of activities” (*β* = 0.23, *p* = 0.049), “location” (*β* = 0.20, *p* = 0.029) also has a significant relationship (Table 3).

**Figure 2:**
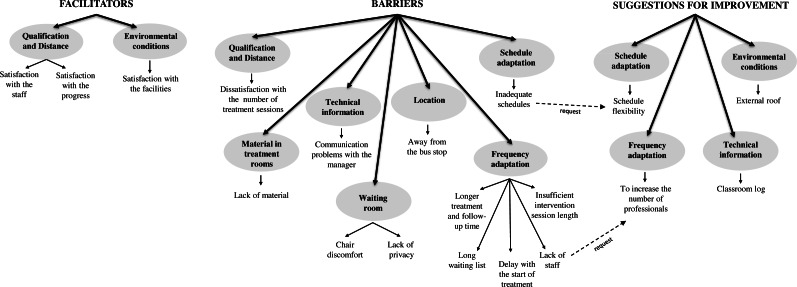
Tree of themes, categories and codes.

Regarding the qualitative analysis, three main themes appeared (facilitators, barriers and suggestions for improvement), as well as eight categories that corresponded to the factors generated from the factor analysis from the quantitative analysis. The tree of themes, categories and codes is shown in Fig. 2.

The facilitators of the service fall, mainly, into the category of *qualification and distance*, within which two codes emerged, with *satisfaction with the staff* being the common one. Many of the participants claimed to be grateful to the staff of the center for the treatment they received from them: (*PA62*) “...*very happy with the treatment received, especially from... (the psychologist) and... (the speech therapist), who are the ones who work with my daughter*”. Moreover, in many cases, and in agreement with the satisfaction with the staff, they highlighted the *satisfaction with the progress* regarding the evolution of the child: (*PV13*) “*...the staff are close and very professional, and the progress of the children here is incredible. I’m very satisfied and grateful*”; (*PA77*) “*...I’m very happy... in the short time we’ve been here I can see the evolution of my son (his self-esteem and confidence when talking is very high)*”. Within the category of *environmental conditions*, there were also references to the *satisfaction with the facilities*: (*PV115*) “*...so far I’m very happy with the location of the center, parking lot...*”.

With respect to the topic of barriers, twelve codes emerged, as can be seen in Fig. 2, which explained the information gathered quantitatively for all the categories, except for *environmental conditions*, where the participants suggested improvements. Next, quotes of the most representative codes are presented. One of the most frequently mentioned categories was *frequency adaptation*, within which five fundamental codes appeared. One of them highlighted the need for *longer treatment and follow-up time*, as stated by PV132: “*...the children should be treated and followed-up beyond the age of 6 years. There are children over 6 years of age who need help. They are working with a professional and, suddenly, they cannot continue that work”.* Likewise, the participants mentioned the *insufficient intervention session length* and the possible particular solutions carried out by the parents: (*PA78*): “...*I think that the attention that my son receives (45 min per week) is not enough to treat his problem, which is why I’m forced to look for a private center in order to help him improve*”. The participants mentioned the *long waiting list* and *lack of staff*: (*PV60*) “*In my humble opinion, I think there is a shortage of staff. There are many children and a long waiting list*”. Based on these barriers, within the topic of improvements, they proposed to *increase the number of professionals*: (*PV120*) “*...increase the number of specialists to meet the current demand*”.

**Table 2:** Sociodemographic characteristics of the participants.

Characteristics	No of responses	Percentage (%)
Gender		
Male	60	25.8
Female	165	70.8
Missing	8	3.4
Age (range of age)		
<20	4	1.7
21–30	46	19.7
31–40	129	55.4
41–50	37	15.9
51–60	4	1.7
>61	1	0.4
Missing	12	5.2
Family relationship		
Mother	155	66.5
Father	59	25.3
Aunt	4	1.7
Uncle	2	0.9
Grandmother	4	1.7
Grandfather	1	0.4
Caregiver	1	0.4
Others	2	0.9
Missing	5	2.1
Academic studies		
Elemental	8	3.4
Primary	79	33.9
Secondary	83	35.6
University	49	21.0
Masters	3	1.3
Professional studies	2	0.9
Missing	9	3.9
Stay in treatment (months)		
6–12	128	54.9
13–24	52	22.3
25–36	19	8.2
37–48	14	6.0
49–60	4	1.7
61–72	1	0.4
Missing	15	6.5

**Table 3:** Linear regression analysis to predict satisfaction and future intention.

	B		95% CI (B)			*β*		*t*-value				*p*-value			
Model 1: *Satisfaction*						
Location		0.07	−0.03–0.18			0.13					1.39				0.167	
Environmental conditions		0.05	−0.12–0.22			0.07					0.58				0.559	
Waiting room		0.02	−0.11–0.16			0.03					0.32				0.747	
Treatment rooms		−0.16	−0.30–−0.02			−0.28		−2.25		0.026	
Material		−0.01	−0.21–0.18			−0.02		−0.15		0.877	
Consumer service		0.01	−0.20–0.22			0.01					0.10				0.920	
Schedule adaptation		−0.02	−0.10–0.06			−0.04		−0.46		0.644	
Frequency adaptation		0.00	−0.08–0.09			0.01					0.06				0.952	
Personnel qualification		−0.05	−0.33–0.23			−0.05		−0.35		0.726	
Personnel coordination		−0.02	−0.22–0.17			−0.03		−0.24		0.811	
Adaptation of activities		0.24	0.04–0.43			0.27					2.34				0.020	
Technical information		−0.07	−0.20–0.05			−0.10		−1.10		0.272	
Model 2: *Future intention*						
Location		0.11	0.01–0.20			0.20					2.19				0.029	
Environmental conditions		0.06	−0.09–0.22			0.09					0.77				0.440	
Waiting room		−0.08	−0.20–0.05			−0.13		−1.23		0.217	
Treatment rooms		−0.05	−0.18–0.08			−0.10		−0.80		0.423	
Material		−0.06	−0.24–0.12			−0.08		−0.65		0.512	
Consumer service		−0.02	−0.21–0.16			−0.02		−0.24		0.811	
Schedule adaptation		−0.04	−0.11–0.04			−0.08		−1.01		0.314	
Frequency adaptation		0.01		−0.07–0.08			0.02	0.20				0.837				
Personnel qualification		0.00		−0.25–0.26			0.01	0.03				0.973				
Personnel coordination	0.00		−0.18–0.18			0.01	0.03				0.972				
Adaptation of activities	0.18		0.00–0.36			0.23	1.97				0.049				
Technical information	−0.05			0.16–0.06			−0.08	−0.88					0.378				

**Notes.**

* *p* < 0.05.

95% CI (B) confidence interval for B *β* standardized B

Regarding the category of *schedule adaptation*, some participants mentioned the *inadequate schedules* of the sessions: (*PV126*) “*Considering that they are children, the schedules should not overlap school hours, to avoid taking them away from school*”. In this sense, the code *schedule flexibility* emerged, as an improvement: (*PV65*) “*It would be convenient to be able to go to the center in the afternoon, in order to find a balance between work and taking our children to receive their treatment*”.

Within the topic of improvements, in addition to the proposals already commented from the detected barriers, within the category of *environmental conditions*, the participants mentioned the possibility of building an *external roof* that protects the users from the weather, especially in the parking lot of the center, when moving from their vehicles to the building and vice versa: (*PV89*) “*They should install a canopy in the parking lot to protect the users from the sun and rain, so that the children do not get hot or wet on their way to and from the center*”. Lastly, they also proposed to have a *classroom log* (in the category of *technical information*), to record the progress of the children: (*PA43*) “*I would like to have a classroom log in which the specialists would write down, every day, the work carried out in the classroom and the valuation of the professional about such work*”.

## Discussion

Throughout this article, the authors have shown the exemplification of a research structure based on a mixed methodology that involves the collection, analysis and interpretation of quantitative and qualitative data in a single study to explore the same objective (Bergman, 2010). In this sense, the logical structure of the scientific method was followed (Anguera et al., 2018). Therefore, this mixed method approach allowed reaching consistency throughout the research process, combining statistics with the real analysis offered by qualitative techniques, using a critical and reflective process. The qualitative data complement the quantitative data, which provided the possibility of obtaining a broader perspective. The measuring model used in this study, which included a scale to evaluate service quality, a scale to evaluate satisfaction and another scale to evaluate future intention, showed adequate psychometric properties with a satisfactory fit in the different indices considered [*χ*^2^(1080) = 1940.48 (*p* < .001); *χ*^2^/gl = 1.79; CFI = 0.91; IFI = 0.90; RMR = 0.042; RMSEA = 0.059 (C.I. = 0.54, 0.63)]. Therefore, it is an advancement with respect to other studies that used models in which none of these three constructs are involved (Giné et al., 2013; Gràcia et al., 2019; Hopwood, 2007; Mas et al., 2018).

In the EIS context there are few studies that specifically evaluate the satisfaction of families with early intervention services (García et al., 2008). Focusing on Spain, according to different studies (Giné, 2002; Giné et al., 2009), the families who use EIS value these services positively and express great satisfaction. However, they also highlight some aspects that could be improved, such as the limited hours of therapy and the termination of the service after 6 years of age. In this sense, this is consistent with the results obtained in this study, since many families express their dissatisfaction with the number of treatment sessions, which are always insufficient in their opinion. Regarding the need to continue with the therapy beyond the age of 6, this is usually a recurrent demand in studies conducted in Spain, since it is at that age when EIS ceases to be provided, in compliance with the guidelines recommended in the EI White Paper (GAT, 2005).

The qualitative results obtained in this study reflect that the participants perceive that the needs of the families attended to in EI centers are not sufficiently satisfied (barriers and suggestions for improvement); they demand, for instance, more sessions, longer schedules, and the possibility to continue with the therapy beyond the age of 6. In this sense, a study showed that a large majority (81%) claimed to be unsatisfied with the attention that the EIS professionals paid to their needs (Baily Jr et al., 2004). However, they were satisfied with the service they provided to their children; these results also support those obtained by another study (Cerqueira, Dessen & Pérez-López, 2012), indicating that the parents evaluated the work of the professionals positively, with this satisfaction with the staff being in line with the results of the qualitative analysis. The results of the present study showed the importance of technical assistance, where the quantitative analysis showed the adaptation of the activities developed by the EIS professionals as a significant factor in the satisfaction model within the regression analysis (*β* = 0.27, *p* = 0.020). The participants highlighted the existence of a long waiting list (waiting times for services) and proposed to increase the number of professionals in order to overcome this barrier, which is a result similar to that found in another study (Horridge et al., 2019). Likewise, they also proposed to install a roof in the parking lot (“environmental conditions”) and implement a classroom log. Considering the opinion of the families, these will participate more actively in the making of decisions, thus their satisfaction with the service provided will increase (Rosenbaum & Gorter, 2011; Law et al., 2011; Ketelaar et al., 2008).

Several authors (Pretis, 2012; Escorcia-Mora et al., 2018) state that intervention quality is clearly affected by the quality of the relationships between the child, the family and the professionals, although in this case it was not a significant aspect within the regression model that explains satisfaction (*β* = 0.01, *p* = 0.920). However, the results of the analysis showed satisfaction with the staff as a facilitator. The regression model that explains future intention offered “location” (*β* = 0.20, *p* = 0.029) and “adaptation of activities” (*β* = 0.23, *p* = 0.049) as the only significant variables, which gives even further meaning to the demands related to the location category regarding the parking issues; this also puts further emphasis on the importance that the activities developed by the EIS professionals have on the therapy given to the children. Some of the improvement propositions of the families are difficult to implement by the EIS, for example, reducing the waiting list to be attended to or continuing to receive treatment in the EIS beyond the 6-year period, since this directly depends on the public administration as well as on the budget assigned by this to EIS. However, other suggestions would be easy to implement by the centers, for example, installing a cover in the parking lots, establishing a classroom log or even making the center attention schedules more flexible. Carrying out these types of studies frequently would allow monitoring the improvement proposals provided by some families, as well as controlling the actions conducted.

The evaluation of the quality of the services provided in EIS centers will allow managers and professionals of such centers to improve the aspects that the families evaluate negatively and strengthen those in which a positive evaluation is obtained. These analyses will facilitate the comparisons with similar services (benchmarking actions). In conclusion, this will improve communication with the users of their services and the planning of different strategies to increase the satisfaction of the clients. Moreover, nowadays it is difficult to conceive a study on a social group in which the people who make up such a group do not participate actively (in this case, the families) (Calabrese & Scoglio, 2012; Torres-Moraga & Lastra-Torres, 2008). The relevance of these results lies in the fact that there are no studies in the literature that evaluate future intention in users of EI centers.

This study is not exempt from limitations. One of the limitations is about the sample of the study. It is a convenience sample, and it comes from the specific geographical context in which the sample was recruited (southern Spain). Therefore, this scale should be used in other Spanish EIS centers to determine whether the results can be replicated, using also another type of statistical analysis, such as Generalizability Theory (Torres-Moraga & Lastra-Torres, 2008), identifying and measuring the variance components that generate estimation errors. This would allow estimating the optimal sample size in the evaluation, as well as the precision of the generalization (Cronbach et al., 1972), which is an adequate and very useful tool for the future planning of studies targeted to the evaluation of services in a more precise and exact manner (Blanco-Villaseñor, 2001). Although in purely qualitative studies this does not pose a problem, this work lacks a more heterogeneous representation of the study population. Nevertheless, the use of the mixed method approach allowed conducting a thorough analysis of the perception of the participants toward EIS quality, which was one of the objectives proposed.

In future research lines related to this material, it would be interesting to relate the degree of satisfaction and the quality perceived by the families with other factors, such as the reason for referral to the center or the pathology by which this was derived, as well as the severity of the latter. Likewise, in future studies, it would be interesting to perform new analyses that relate variables such as the gender or age of the main caregivers.

## Conclusions

In conclusion, knowing the perception of the families toward the quality of the service provided in EI centers from a research perspective based on quantitative and qualitative analyses, allows us to successfully coordinate the objectives of both users and professionals. The development of qualitative studies provide complementary evidence (Bradbury et al., 2015), and on the other hand, it is worth highlighting the importance of involving the families-users in the EI programs (Ebbels et al., 2019). Active participation makes the families feel as if they are part of the treatment, offering the possibility to communicate their perceptions beyond the items of a questionnaire is a very positive aspect.

##  Supplemental Information

10.7717/peerj.10193/supp-1 Supplemental Information 1 COREQ Checklist

10.7717/peerj.10193/supp-2 Supplemental Information 2 Raw data-IQEIC-Mixed Methods-English

10.7717/peerj.10193/supp-3 Supplemental Information 3 Inventory of Quality in Early Intervention Centres

## Additional Information and Declarations

### Data Availability

The following information was supplied regarding data availability: Participant responses to Inventory of Quality in Early Intervention Centres (IQEIC) are available in the Supplemental Files.
