# Supplementary material for: What families really think about the quality of early intervention centers: a perspective from mixed methods
Source: PeerJ. 2020 Oct 20;8:e10193. doi: 10.7717/peerj.10193 (PMC7583605; doi:10.7717/peerj.10193)
Supplement: Supplemental Information 3 [file peerj-08-10193-s003.pdf]

INVENTORY OF QUALITY IN EARLY INTERVENTION CENTRES

We are interested in doing a QUALITY ASSESSMENT IN EARLY INTERVENTION CENTRES. Your opinion is very important to improve the quality of service of our centre. For this reason we would appreciate you answering the following questionnaire.

Please, indicate the number that is closest to the valuation that you want to indicate, for example, if you "strongly agree", indicate box 5 or, if on the contrary, "do not agree", indicate box 1 in the statements listed below. There are no bad or good answers, true or false, we only WANT TO KNOW YOUR SINCERE OPINION ABOUT OUR SERVICE. The data contained herein will be confidential and will only be used for research purposes. Thank you very much for your help.

**Do not agree**				**Strongly agree**
1	2	3	4	5

**1: Facilities of the centre**	**1**	**2**	**3**	**4**	**5**
1. The centre is well located geographically					
2. It is easy to reach the centre by public transport					
3. The directions to get to the centre are sufficient					
4. The centre has removed the architectural barriers					
5. The cleaning of the centre is adequate					
6. The lighting of the centre is adequate					
7. The ventilation of the centre facilities seems correct					
8. The temperature of the centre is pleasant					
9. The centre has toilets adapted to the user´s needs					
10. The centre has waiting room					
11. The waiting room is comfortable					
12. The number of chairs in the waiting room is enough					
13. The waiting room has playful material					
**2: Treatment rooms and material**	**1**	**2**	**3**	**4**	**5**
14. The number of treatment rooms is enough					
15. The treatment rooms are large enough					
16. The temperature of the treatment rooms is nice					
17. The ventilation of the treatment rooms is correct					
18. The materials that are used in the center are suitable					
19. The centre has enough material to work with your child					
20. The materials are in good condition for use					
21. The materials that are used in the treatment rooms are safe					
22. The work materials comply with the health and hygiene conditions					
**3: Specific sessions**	**1**	**2**	**3**	**4**	**5**
23. The attention that users receive at the centre is suitable					
24. The number of treatment sessions per week is sufficient					
25. The opening hours are adapted to your needs					
26. You have been given a schedule change when requested					
27. The schedule assigned by the centre is usually respected					

	1	2	3	4	5
28. When your child improves or worsens, the centre change the frequency of treatment					
29. When for any reason, they cancel an appointment, they notify you with sufficient time					
**4: Specialized staff**	**1**	**2**	**3**	**4**	**5**
30. Qualified staff have the necessary knowledge					
31. Qualified staff are accesible					
32. Qualified staff are available when users need them					
33. Qualified staff have a close treatment					
34. Your child is comfortable with the qualified staff .					
35. I value the contributions and initiatives of the qualified staff					
36. Qualified staff are coordinated among themselves					
37. Qualified staff know to adapt tasks to the user's needs					
**5: Technical assistance**	**1**	**2**	**3**	**4**	**5**
38. The activities carried out with the user seem appropriate					
39. The activities proposed for users to work on at home are feasible					
40. The information received at the beginning of the treatment is consistent with the tasks subsequently performed					
41. I usually receive some programs to work with the user					
42. I usually receive some report about the progression of the user					
43. The information received about the user is clear					
**6: Satisfaction**	**1**	**2**	**3**	**4**	**5**
44. I am satisfied with the services of this Early Intervention Centre (EIC)					
45. I am satisfied with my decision to join this Early Intervention Centre (EIC)					
46. I am pleased to have taken the decision to come to this Early Intervention Centre (EIC)					
**7: Future intention**	**1**	**2**	**3**	**4**	**5**
47. I will make positive comments to a friend about the programs and services of this EIC					
48. If you ask me, I will recommended this EIC					
49. I will continue to participate in the programs and services of this EIC					
50. I would continue in this EIC if I had the need again					

**Sociodemographic data**		**Kinship with the child (mark one)**	
**Early Intervention Centre City:**			
**Genre: M (1. Man) W (2. Woman)**		**Mother:**	
**Age:**		**Father:**	
**Place of birth:**		**Aunt:**	
**No studies:**		**Uncle:**	
**Primary studies:**		**Grandmother:**	
**Mid-level studies:**		**Grandfather:**	
**Higher Education:**		**Caregiver :**	
**Postgraduate Studies:**		**Other:**	
**Others:**			
**Time since the beginning of the Early Intervention treatment (months):**			
**Have you previously visited another centre?**			

**Please, write an observation or suggestion:**
